# Decreased Bone Formation and Osteopenia in Lamin A/C-Deficient Mice

**DOI:** 10.1371/journal.pone.0019313

**Published:** 2011-04-25

**Authors:** Wei Li, Li Sze Yeo, Christopher Vidal, Thomas McCorquodale, Markus Herrmann, Diane Fatkin, Gustavo Duque

**Affiliations:** 1 Ageing Bone Research Program, Nepean Clinical School, University of Sydney, Penrith, New South Wales, Australia; 2 Victor Chang Cardiac Research Institute, Darlinghurst, New South Wales, Australia; 3 Cardiology Department, St Vincent's Hospital, Darlinghurst, New South Wales, Australia; 4 Faculty of Medicine, University of New South Wales, Kensington, New South Wales, Australia; Institut de Génomique Fonctionnelle de Lyon, France

## Abstract

Age-related bone loss is associated with changes in bone cellularity with characteristically low levels of osteoblastogenesis. The mechanisms that explain these changes remain unclear. Although recent *in vitro* evidence has suggested a new role for proteins of the nuclear envelope in osteoblastogenesis, the role of these proteins in bone cells differentiation and bone metabolism *in vivo* remains unknown. In this study, we used the lamin A/C null (*Lmna* ^−/−^) mice to identify the role of lamin A/C in bone turnover and bone structure *in vivo*. At three weeks of age, histological and micro computed tomography measurements of femurs in *Lmna* ^−/−^ mice revealed a significant decrease in bone mass and microarchitecture in *Lmna* ^−/−^ mice as compared with their wild type littermates. Furthermore, quantification of cell numbers after normalization with bone surface revealed a significant reduction in osteoblast and osteocyte numbers in *Lmna* ^−/−^ mice compared with their WT littermates. In addition, *Lmna* ^−/−^ mice have significantly lower osteoclast number, which show aberrant changes in their shape and size. Finally, mechanistic analysis demonstrated that absence of lamin A/C is associated with increase expression of MAN-1 a protein of the nuclear envelope closely regulated by lamin A/C, which also colocalizes with Runx2 thus affecting its capacity as osteogenic transcription factor. In summary, these data clearly indicate that the presence of lamin A/C is necessary for normal bone turnover *in vivo* and that absence of lamin A/C induces low bone turnover osteopenia resembling the cellular changes of age-related bone loss.

## Introduction

Age-related bone loss starts after the third decade of life as a consequence of changes in both hormone levels and bone cell differentiation and function [1]. In contrast to bone loss associated with estrogen deficiency, which is explained by increasing levels of osteoclastic activity, the predominant change in age-related bone loss is a switch in mesenchymal stem cells (MSC) differentiation from a predominant osteogenic phenotype in young bone into an adipogenic phenotype in old bone [2], thus affecting bone formation and bone quality [3]. The mechanisms explaining this predominant differentiation of MSC into adipocytes in aging bone remain to be elucidated.

Amongst the potential mechanisms linking osteoporosis and the cellular aging process, the role of the proteins of the nuclear envelope in general and lamin A/C in particular is a subject of increasing interest [4]. Lamins are intermediate filament proteins present in the nuclear lamina and matrix that are critical determinants of nuclear architecture and are important regulators of stem cells differentiation [5], [6]. The lamin family in mammals includes 7 different proteins (A, AΔ10, C, C2, B1, B2, and B3). Most adult mammalian somatic cells contain the three major lamins A, B1 and C. These various forms are grouped into two classes, A-type (A, AΔ10, and C) and B-type (B1 and B2). While B-type lamins are found in all nucleated somatic cells, the expression of A-type lamins, which result from alternative splicing events of the lamin A (*Lmna*) gene, is developmentally regulated [5]. A-type lamins are absent from all pre-implantation stage embryonic cells with their synthesis commencing at about day 9 within the visceral endoderm and trophoblast. Subsequently, A-type lamins appear asynchronously in various tissues [5]. In bone, recent evidence has linked the deficiency of processed lamin A/C with the cellular features of senile osteoporosis *in vitro* [7], [8] and *in vivo* [9], [10], suggesting that lamin A/C could play an important role in the pathogenesis of age-related bone loss.

We have recently tested the effect that absence of lamin A/C may have on the differentiation of MSC into osteoblasts [7]. Using a silencing RNA (siRNA) approach, we successfully knocked down lamin A/C in a model of osteoblastogenesis. We found that lamin A/C inhibition affects osteoblastogenesis while favors adipogenesis *in vitro*. Furthermore, in an *in vivo* model of autosomal dominant Enery-Dreifuss muscular dystrophy (EDMD2) mouse obtained due to a missense L530P variant that affects lamin A/C expression, Mounkes et al. [9] reported that homozygous L530P/L530P animals developed features of progeria that included significantly lower bone mineral density (BMD) as compared with their heterozygous and wild type (WT) counterparts. However, the mechanisms of bone loss and the potential association between lamin A/C deficiency and senile osteoporosis have not been fully explored in a lamin A/C null animal model.

In the present study, we further assessed the effect of lamin A/C knockout *in vivo* on bone microarchitecture, osteoblast differentiation and bone turnover using lamin A/C (*Lmna* ^−/−^) mice. This model was developed by deleting exons 8–11 of the *Lmna* gene, which disrupts both isoforms (A and C), shows a dystrophic condition related to EDMD2, including the appearance of skeletal and cardiac muscle alterations and perturbations of the nuclear envelope [11]. At birth, *Lmna* ^−/−^ mice are identical to their heterozygous and WT controls. After birth, homozygous (*Lmna* ^−/−^) mice show accelerated features of aging and have a shorter life span (∼6 weeks) than their heterozygous counterparts. The most common cause of death in *Lmna* ^−/−^ mice is cardiovascular complications due to atherosclerosis and coronary artery disease [12].

Our results demonstrate that *Lmna* ^−/−^ mice are severely osteopenic and show low levels of bone turnover associated with a decrease in bone formation that exceeds the decrease in bone resorption, clearly mimicking the cellular changes of senile osteoporosis. In addition, a mechanism explaining the decline in osteoblastogenesis in *Lmna* ^−/−^ mice is proposed. Taken together, these data confirms previous *in vitro* data on the role of lamin A/C in osteoblastic differentiation of MSC differentiation and opens the path for the development of anabolic drugs that maintain the bone mass through the regulation of lamin A/C expression and its interaction with other nuclear proteins in differentiating MSC.

## Results

### Absence of Lamin A/C Affects Bone Mass and Microarchitecture

**Figure 1:**
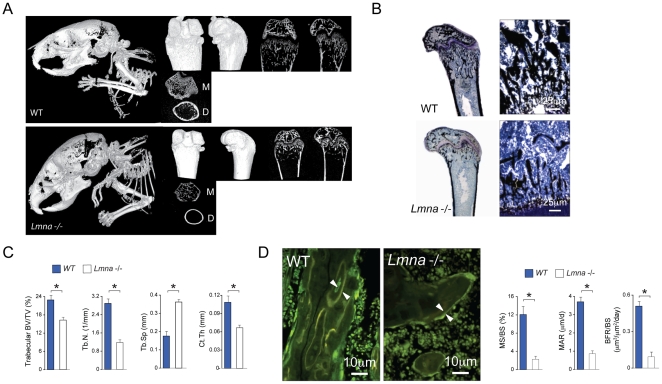
Changes in bone architecture in *Lmna* ^−/−^ mice. (**A**) Micro-CT analysis of total body (left panels) and femur of 4-week-old *Lmna* ^−/−^ mice and WT littermates. Images representative of two-and three- dimensional reconstructions, obtained with a 0.9° rotation between frames on a Skyscan® 1072 instrument, are shown for WT mice (A, upper panel) and *Lmna* ^−/−^ (A, lower panel) mice. Whole-body CT analysis of *Lmna* ^−/−^ mice and WT littermate controls shows dramatically lower bone accumulation in *Lmna* ^−/−^ mice with remarkable skull defects observed in the mutant mice. The right upper panels are representative of longitudinal sections. The lower right panels show the metaphysis (area just below the growth plate) (M) and distal diaphysis (cortical structure) (D). *Lmna* ^−/−^ mice exhibited profound thinning of cortical bone, a reduction in platelike structures and a lack of trabecular connectivity. These changes correlated with von Kossa staining (**B**). Quantitation of bone parameters (**C**) further exemplified a decrease in bone volume vs. total volume (BV/TV), trabecular number (Tb.N), and cortical thickness (Ct.Th) with a concomitant increase in trabecular separation (Tb.Sp) in the mutant femora compared with the WT littermate controls. Results are expressed as the mean ± SD of eight independent analyses per group. *P<0.001, significantly different from null mice. (**D**) Tetracycline-labeled section of the distal femur. The distance between two layers of tetracycline labels (*arrows*) visualized by epifluorescence represents bone formation that occurred during the 5-d period between tetracycline injections. Mutant mice showed a significant decrease in all parameters of bone formation including mineralized surface/bone surface (MS/BS), mineral apposition rate (MAR) and bone formation rate/bone surface (BFR/BS) as compared with their WT littermates (*P<0.001). Photomicrographs were obtained on the Bioquant analysis system using a ×40 objective.

Using *Lmna* ^−/−^ mice [11], we analyzed whether absence of lamin A/C has an effect on bone mass and microarchitecture. Figure 1 shows a qualitative (A, B) and quantitative (C) decline in bone density and architecture in *Lmna* ^−/−^ mice as compared with wild type (WT) controls. Whole body analysis of *Lmna* ^−/−^ mice and WT littermate controls (Figure 1A, left panels) shows dramatically lower bone mass in the mutants as compared WT controls. In addition, the skull of the mutants show the typical defects previously reported by Mounkes et al [9]. No significant difference in either long bone size or diameter was found between *Lmna* ^−/−^ mice as compared with wild type (WT) controls (data not shown). 3D reconstruction and 2D saggital histological sections of the distal femur (Figure 1A) showed a significant loss in trabecular and cortical bone in *Lmna* ^−/−^ mice as compared with the WT controls. Histological and micro-computed tomography (CT) measurements of distal femur in *Lmna* ^−/−^ mice revealed a significant decrease in bone volume/trabecular volume (BV/TV), trabecular number (Tb.N), and cortical thickness (Ct.Th) with a significant increase in trabecular separation (Tb.Sp) (Figure 1C), compared with their WT littermates (*P*<0.001). Furthermore, levels of mineral apposition identified by fluorochrome labelling on two separate dates (day 5 and 2 pre-sacrifice), showed mineralized surface per bone surface (MS/BS) (WT, 12%; *Lmna* ^−/−^, 2%, *P*<0.001) and mineral apposition rate (MAR) decreased 4-fold, and the rate of bone formation rate per bone surface BFR/BS decreased by approximately 10-fold (WT, 0.5 µm^3^/µm^2^·d; *Lmna* ^−/−^, 0.05 µm^3^/µm^2^/d, *P*<0.001) (Figure 1D). Taken together these data indicate that absence of lamin A/C *in vivo* is associated with severe osteopenia, poor bone microarchitecture and significantly lower levels of bone apposition.

### Analysis of Osteoblast Differentiation and Activity

**Figure 2:**
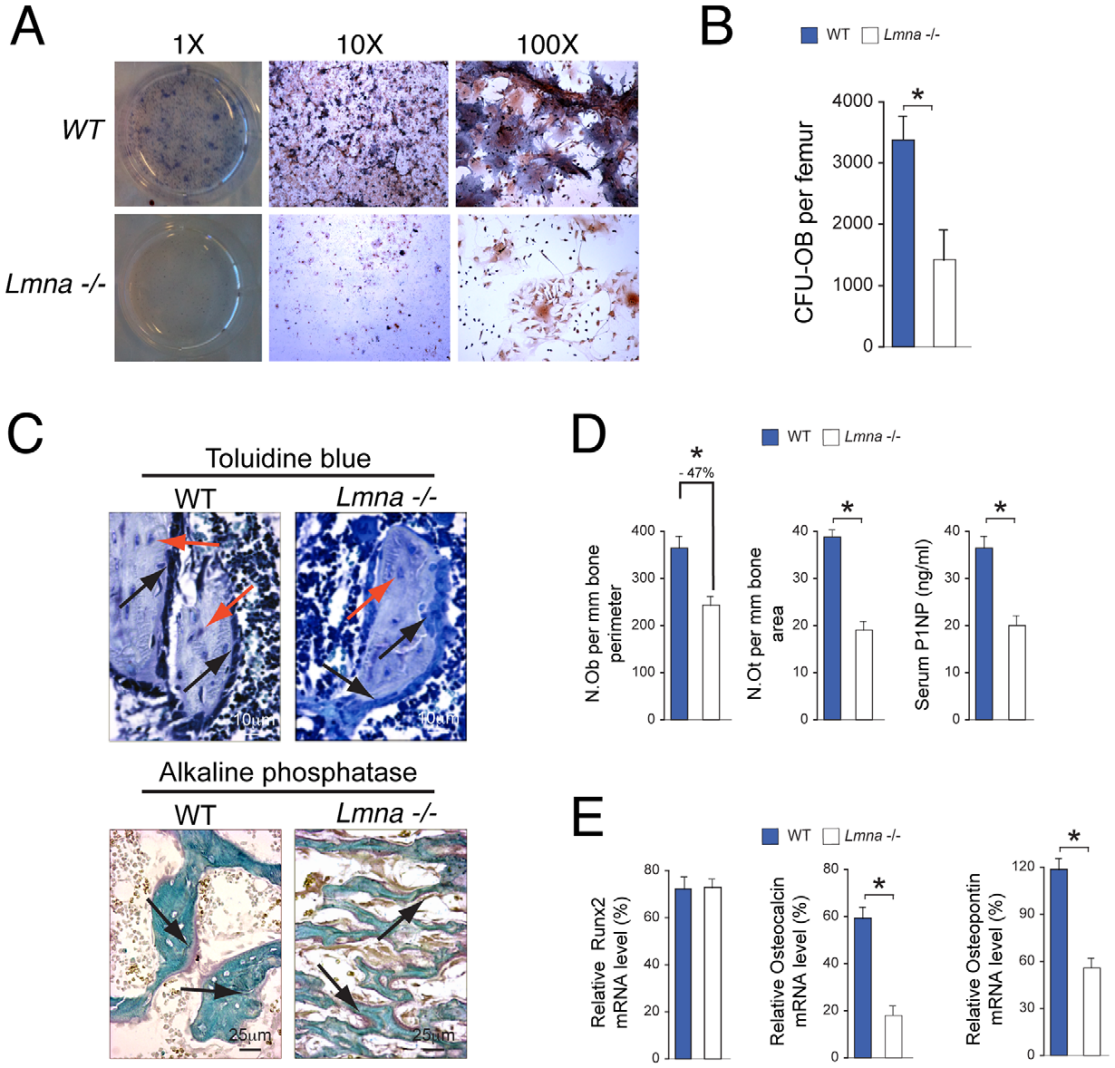
Changes in osteoblast differentiation and function of 4 week-old *Lmna* ^−/−^ mice. (**A and B**) Formation of colony forming units-osteoblasts (CFU-OB) in ex-vivo cultures of bone marrow cells from 3-week-old *Lmna* ^−/−^ mice (lower panels) and WT controls (upper panels). The number of colony forming units-osteoblast (CFU-OB) per femur was significantly higher after 3 wks of differentiation in WT mice compared to *Lmna* ^−/−^ mice (**B**). (**C**) Sections of plastic embedded proximal tibiae (secondary spongiosa) from *Lmna* ^−/−^ mice and WT controls (n = 10 per group) were stained sequentially with toluidine blue (upper panels, Magnification ×40) for osteoblasts (black arrows) and osteocytes (red arrows) and ALP (lower panels, Magnification ×20) for osteoblasts (arrows). (**D**) A significant decrease (−47%) in the number of ALP expressing osteoblasts (N.Ob) and a significant decrease in osteocyte number (N.Ot) (−50%) were seen in *Lmna* ^−/−^ mice compared with WT^+/+^ mice. Micrographs are representative of those from eight different mice of each genotype. *P<0.001. Changes in osteoblast differentiation and function correlated with changes in a serum biochemical marker of bone formation (P1NP) in *Lmna* ^−/−^ mice compared with WT^+/+^ mice. *P<0.001. (**E**) The reduction in osteoblast differentiation in *Lmna* ^−/−^ mice was associated with lower expression of osteocalcin (OCN) and osteopontin (OPN) at the mRNA level. There were no differences in Runx2 expression between *Lmna* ^−/−^ mice and their WT controls. For PCR, data analysis is expressed as the ratio of the gene of interest vs. GAPDH as control. Data represent the mean±SD of triplicate determinations. *P<0.001.

To determine whether the structural changes in bone and the low bone formation rate observed in *Lmna* ^−/−^ mice are associated with a deficit in bone forming osteoblasts, analysis of osteoblastic differentiation of pluripotent bone marrow cells and osteoblast number and activity in bone tissue were performed. After three weeks in osteogenic media, the number of osteoblast precursors obtained from bone marrow and represented by colony forming units – osteoblasts (CFU-OB) was significantly lower in *Lmna* ^−/−^ mice as compared with their WT controls (Figure 2A and B, *P*<0.001). Furthermore, quantification of cell numbers after normalization with bone surface revealed a significant reduction in osteoblast and osteocyte numbers (Figure 2C and D) in *Lmna* ^−/−^ mice compared with their WT littermates (*P*<0.001). Furthermore, in agreement with the differences in osteoblast number, serum levels of procollagen type 1 amino terminal propeptide (P1NP) (Figure 2D) were significantly lower in *Lmna* ^−/−^ mice compared with their WT littermates (*P*<0.001).

Finally, we determined whether the reduction in osteoblast differentiation was associated with changes in the expression of markers of osteoblast differentiation at the mRNA level (Figure 2E). We found a significant reduction in the expression levels of mRNA for osteocalcin (OCN) and osteopontin (OPN) in *Lmna* ^−/−^ mice as compared with their WT controls (*P*<0.001). In contrast, there were no differences in mRNA levels of runt-related transcription factor 2 (Runx2), a critical factor for osteoblastogenesis and an upstream regulator of OCN and OPN transcription [13].

### Analysis of Osteoclast Number and Activity

**Figure 3:**
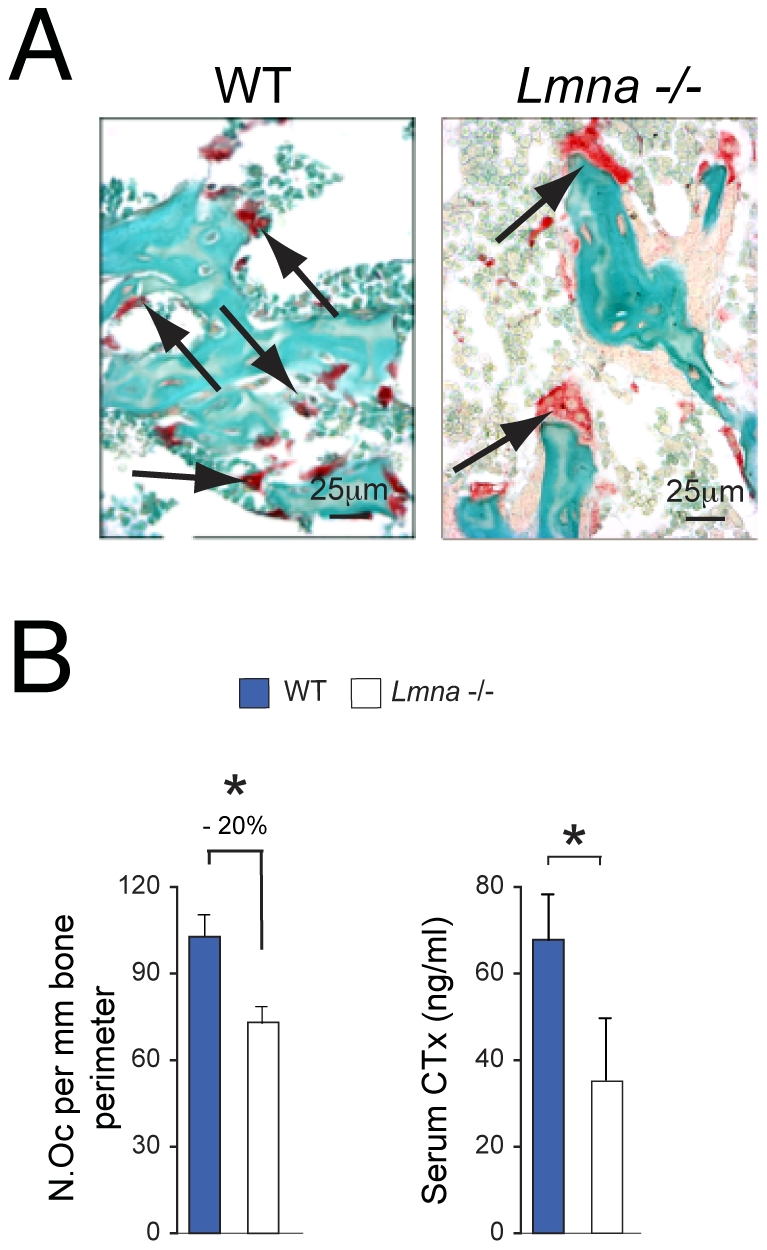
Changes in osteoclast number and function of 4 week-old *Lmna* ^−/−^ mice. (A) Sections of plastic embedded proximal tibiae from *Lmna* ^−/−^ mice and WT (n = 10 per group) were stained sequentially for TRAP (osteoclasts, OC) (arrows, Magnification ×40). A significant decrease in the number of OC (N.Oc) showing TRAP enzyme activity (B) was seen in *Lmna* ^−/−^ mice compared with WT controls. In addition, osteoclasts in the *Lmna* ^−/−^ mice showed an aberrant phenotype including giant size and vacuolization (arrows). Micrographs are representative of those from eight different mice of each genotype. *p<0.001. (**B**) Changes in bone cellularity correlated with changes in serum biochemical markers of bone resorption (CTx: C-telopeptides) in *Lmna* ^−/−^ mice compared with WT controls. *P<0.01.

To determine whether, as in the osteoblast lineage, osteoclasts are affected by absence of lamin A/C, we stained decalcified sections of tibiae with tartrate resistant acid phosphatase (TRAP) to identify osteoclasts (Figure 3A). Quantification of cell numbers after normalization with bone surface (Figure 3B) revealed a significant reduction in osteoclast number in *Lmna* ^−/−^ mice compared with their WT littermates (*P*<0.001). The decrease in osteoclast number observed in *Lmna* ^−/−^ mice (∼20%) was significantly lower than the decrease induced by absence of lamin A/C on osteoblast number (∼47%, *P*<0.001) (Figure 2C). In addition, osteoclasts in the *Lmna* ^−/−^ mice showed phenotypic abnormalities including big size and vacuolization (Figure 3A).

Finally, to determine whether the reduction in cell numbers seen in *Lmna* ^−/−^ mice correlated with circulating levels of biomarkers of osteoclastic activity (bone resorption) similar to those used in the clinical setting, we measured serum levels of C-telopeptide (C-Tx) in both null and WT animals. Figure 3B shows a significant decrease in serum C-Tx in *Lmna* ^−/−^ mice as compared with their WT controls (*P*<0.01), also correlating with the lower levels of osteoclast numbers found in the histochemical analysis.

### Circulating Concentrations of Calciotropic Hormones in *Lmna^−/−^* mice

To determine whether the changes in bone cellularity and microarchitecture were due to alterations in serum concentrations of calciotropic hormones, we measured serum levels of parathyroid hormone (PTH) and 25-dihydroxy-vitamin D [25(OH)D] in *Lmna* ^−/−^ mice and compared them with their WT controls. As shown in table 1, there were no differences in serum levels of either PTH or 25(OH)D between *Lmna* ^−/−^ mice and WT controls.

**Table 1:** Circulating concentrations of calciotropic hormones parathyroid hormone (PTH) and 25(OH)-vitamin D [25(OH)D] in *Lmna* ^−/−^ mice vs. WT controls.

*Assay*	*WT*	*Lmna^−/−^*	*P value*
	N = 10	N = 10	
**25(OH)D (nmol/l)**	48±14	60±15	NS
**PTH (pg/ml)**	26±6	29±5	NS

### Analysis of Nuclear Protein and Nuclear Factors Interaction in Bone Cells of *Lmna^−/−^* Mice

**Figure 4:**
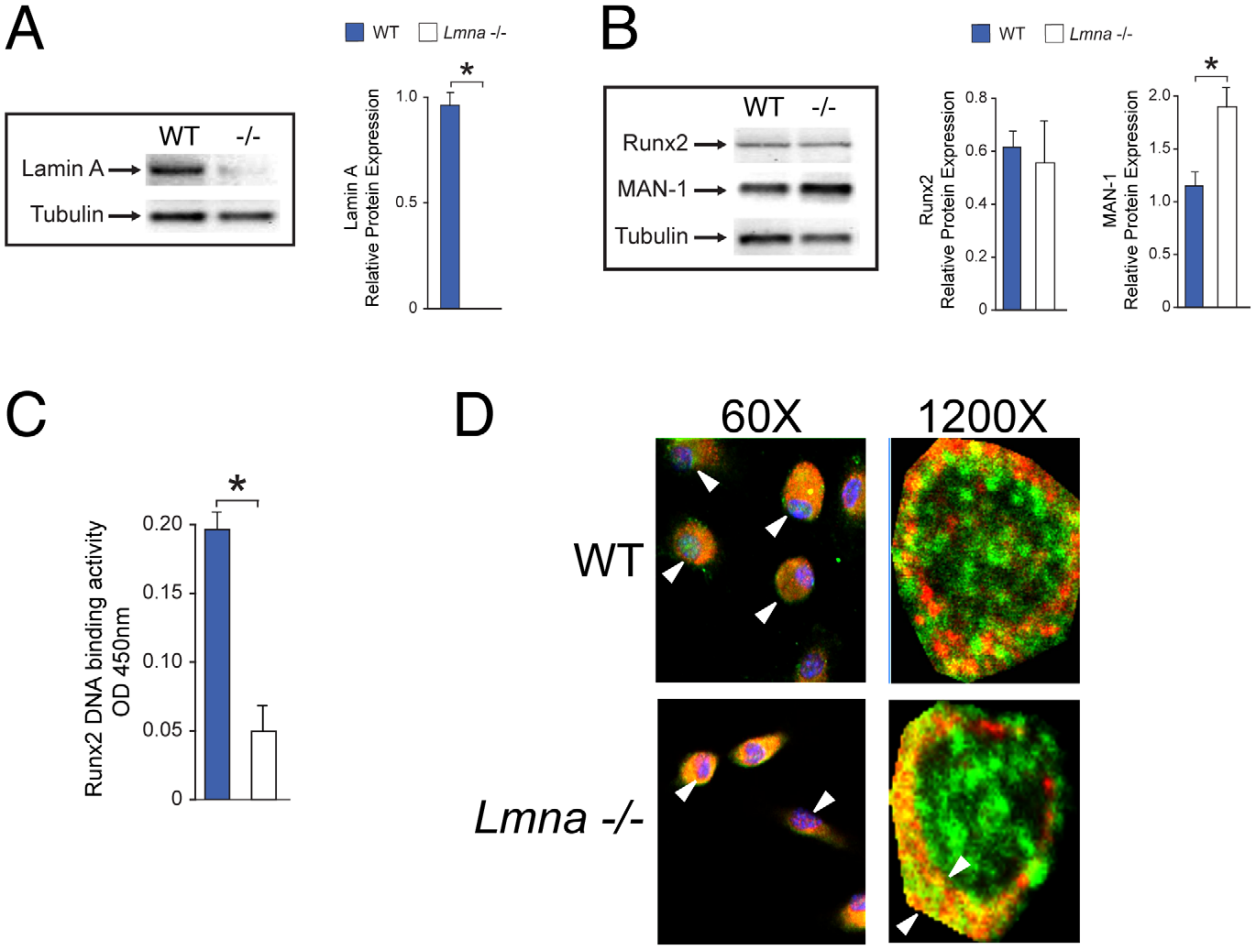
Mechanism of bone loss in *Lmna* ^−/−^ mice. (**A and B**) Expression of lamin A is abolished in *Lmna* ^−/−^ mice as compared with their WT controls. (*P<0.001) (A). Furthermore, no difference in protein levels of Runx2 were identified in protein extracts from *Lmna* ^−/−^ mice vs. WT control (B). Finally, expression levels of MAN-1 were significantly higher in *Lmna* ^−/−^ mice as compared with their WT counterpart (B). Protein levels relative to tubulin were quantified by densitometry. Data represent the mean±SD of triplicate determinations. *P<0.001. (**C**) Bone marrow cells obtained from *Lmna* ^−/−^ mice showed a significant reduction in Runx2 DNA binding activity as compared with WT controls (*P<0.01). The data are representative of three different experiments. (**D**) Confocal microscopy of nuclei of marrow precursors (passage 2) obtained from tibiae of *Lmna* ^−/−^ and WT controls and cultured *ex vivo* in MSC growth media for 3 days. In cells obtained from WT mice, Runx2 (green) is widely distributed in the nucleus (upper panels, white arrows) whereas MAN-1 (red) distribution is mostly limited to the nuclear envelope. In contrast, in absence of lamin A/C (lower panels), both Runx2 and MAN1 share the same nuclear peripheral colocalization with extremely low levels of Runx2 expression (green) seen in the interior of the nuclei. Note also the smaller nuclei in MSC obtained from the mutants. DAPI (blue) was used as counterstaining only at lower magnification (left panels). In right panels, Arrows denote size of colocalization (right panels). Images are representative of cell cultures from 6 different mice.

Initially, the expression of lamin A protein in bone marrow cells was determined by western blot (Figure 4 A and B). As expected, expression of lamin A protein was abolished in the *Lmna* ^−/−^ mice as compared with their WT littermates (Figure 4A). Subsequently, using nuclear extracts obtained from non-hematopoietic, adherent stromal cells we assessed the levels of Runx2 protein expression (Figure 4B). We found that, as in our *in vitro* [7] and PCR data, levels of expression of Runx2 protein are not affected by absence of lamin A/C.

Our next step was to determine whether, as reported in our *in vitro* analysis [7], lamin A/C is required for the successful nuclear binding and thus the transcriptional activity of Runx2. Under osteogenic conditions, phosphorylated nuclear Runx2 interacts with a group of proteins known as Smads. The complex Runx2/Smad factors will then bind to the DNA to activate the transcription of some of the most important osteogenic factors such as osteocalcin (OCN) and osteopontin (OPN). To characterize whether Runx2 nuclear binding activity was affected by absence of lamin A/C we used ELISA analysis of Runx2 complex binding. Our analysis showed a significant reduction in Runx2 DNA binding activity in *Lmna* ^−/−^ mice as compared with WT controls (*P*<0.001; Figure 4C).

Since alterations in Runx2 complex binding capacity is a recurrent finding in absence of lamin A/C both *in vitro* [7] and *in vivo*, we therefore hypothesize that this finding could be explained by changes in the interaction between proteins of the nuclear envelope, which have been previously associated with alterations in cell differentiation in other cell models [14]. To assess this hypothesis, we selected the integral inner nuclear membrane MAN-1. This protein physically interacts with lamin A/C, which also acts as a regulator of MAN-1 interaction with other nuclear proteins [15]. In addition, MAN-1 plays an important inhibitory role to the interaction between Smads and other intranuclear proteins [16], [17]. Initially we assessed the changes in MAN-1 protein expression in bone marrow cells of *Lmna* ^−/−^ and WT mice. Expression levels of MAN-1 were significantly higher in nuclear extracts obtained from marrow cells of *Lmna* ^−/−^ mice as compared with WT controls (Figure 4B, *P*<0.01). We then assessed the changes in nuclear interaction between Runx2 and MAN-1 in the absence of lamin A/C by using *ex vivo* cultures of MSC obtained from mutant and WT mice. As shown in Figure 4D, MSC obtained from *Lmna* ^−/−^ mice showed smaller nuclei and lower levels of nuclear MAN-1/Runx2 colocalization as compared with their WT controls.

## Discussion

The present study demonstrates that lamin A/C is required in osteoblastogenesis and bone formation *in vivo*. Additionally, mice lacking lamin A/C showed a decrease in bone formation that exceeds a decrease in bone resorption resulting in significant bone loss mimicking the cellular changes observed in senile osteoporosis [18]. In addition, absence of lamin A/C is associated with 1) alterations in the nuclear transcription required for osteoblastogenesis and; 2) abnormal interaction between Runx2 and MAN-1, a protein of the nuclear envelope that is closely regulated by lamin A/C through a direct physical interaction [14].

The importance of proteins of the nuclear envelope in bone biology has been reinforced by changes in bone mass seen in patients suffering of mutations that either decrease or increase bone mass. For instance, a type of progeria known as Hutchinson Gilford Progeria Syndrome (HGPS) has been associated with mutations in the lamin A/C gene [19]. Patients suffering from HGPS show major bone changes including severe osteoporosis, osteolysis, bone deformities and spontaneous fractures [20]. In contrast, mutations in MAN-1 result in three autosomal dominant diseases in humans known as melorheostosis; all three characterized by increased endochondral and intramembranous bone formation and abnormally high BMD [16].

In the particular case of lamin A/C there is enough evidence to suggest that this protein plays an important role in bone metabolism. Considering that in normal physiological conditions lamin A/C has been associated with telomerase maintenance [21] and stem cell homeostasis [22], the aging process in tissues that depend on normal MSC differentiation could be also associated with changes in lamin A/C expression. This could be the case of bone, where normal osteoblasts show a decrease in lamin A expression with aging [23].

Furthermore, the link between lamin A/C and age-related bone loss is most likely based on the role played by lamin A/C in the differentiation of MSC. It has been proposed that nuclear proteins, such as lamin A/C, may act as signalling receptors in the nucleus required for receiving and/or transducing upstream cytosolic signals in a number of pathways central to adult stem cell maintenance [22]. Indeed, alterations in either lamin A/C processing or expression have been associated with abnormal differentiation of MSC, which may explain the defects in bone (osteopenia) [20] and muscle (dystrophy) [11] observed in patients suffering from severe laminopathies. In terms of processing, lamin A is the end product of prelamin A after exposed to protein cleavage and prenylation [5]. Accumulation of unprenylated prelamin A negatively regulates adipogenic [24] and osteogenic [25] differentiation of MSC *in vitro*. Furthermore, mice lacking the enzyme responsible for lamin A/C processing (Zmpste24^−/−^) show accelerated bone loss and the typical features of senile osteoporosis [10].

For the purposes of the present study, we assessed the changes in bone metabolism and microarchitecture in the lamin A/C null (*Lmna* ^−/−^) mice. Low levels of lamin A/C have been associated with low BMD measured by densitometry in mice [9]. Interestingly, in their mouse model heterozygous mice showed no difference in BMD as compared with WT animals. For this reason, we focused our bone phenotype assessment to *Lmna* ^−/−^ mice and their WT controls.

Initially, we performed both microCT and histological analysis of bones of *Lmna* ^−/−^ mice at the age of 3 to 4 weeks while the animals were still in good health and mobility. This time point was selected due to the short life span of the homozygous mice and to prevent any impact of comorbidities or limited mobility on bone metabolism. Microarchitectural analysis demonstrated that bone mass is significantly lower in the *Lmna* ^−/−^ mice compared to WT control mice, leading to an osteopenic phenotype. This bone loss is reflected by a significant decrease in trabecular number with a concomitant increase in trabecular separation as well as a much lower cortical thickness in *Lmna* ^−/−^ mice, a key feature in age-related bone loss [26]. Furthermore, to analyze the cellular mechanisms of bone loss in this model, we assessed the changes in bone cellularity in *Lmna* ^−/−^ mice as compared to their WT littermates. We found that the number of osteoblasts and osteoclasts relative to the bone volume was significantly reduced in *Lmna* ^−/−^ mice as compared with the WT controls. This reduction in cell number corresponded with lower concentrations of serum markers of osteoblastic (P1NP) and osteoclastic (C-telopeptide) activities. In addition, these changes in bone cellularity were not associated with changes in serum levels of calciotropic hormones. In agreement with other reported models of senile osteoporosis [27], [28], in *Lmna* ^−/−^ mice the reduction in bone formation significantly exceeded the decrease in bone resorption thus becoming a new proposed model of reduced turnover and senile osteoporosis.

The lower number of osteoclasts found in our *in vivo* model are in disagreement with a previous report from Rauner et al [8] in which absence of lamin A/C *in vitro* was associated with high levels of RANKL-induced osteoclastogenesis. The divergence could come from their *in vitro* model where combined osteoblasts and osteoclasts are grown in a well-regulated environment whereas in the *in vivo* model the number of variables involved in the osteoblast-osteoclast interaction is substantial. Indeed, the decrease in osteoclast number and activity observed in *Lmna* ^−/−^ mice could be due to either lower number of osteoblast precursors, which are the major source of RANK-L in bone, or to the aberrant osteoclast phenotype observed in *Lmna* ^−/−^ mice. Nevertheless, the role of lamin A/C in osteoclast differentiation should be the subject of future studies.

Furthermore, considering that osteocytes are the most abundant cell in bone, act as mechanosensors and constitute the final fate of a proportion of osteoblasts [29] and that a recent study associated lamin A/C with mechanosensing and matrix formation [30], we looked at the effect that lack of lamin A/C activity may have on osteocytes number in this model. We found a significant reduction in trabecular osteocyte number in *Lmna* ^−/−^ mice as compared to their WT controls. More studies looking at the role of proteins of the nuclear lamina in osteocyte function and mechanotransduction are required.

As in our *in vitro* experiments [7] the significant reduction in osteoblast number in *Lmna* ^−/−^ mice was associated with lower levels of MSC differentiation into osteoblasts in absence of lamin A/C. In agreement with this evidence, in the present study non-hematopoietic adherent marrow precursors obtained from *Lmna* ^−/−^ mice showed significantly lower capacity to differentiate into osteoblasts under osteogenic conditions. Overall, from the point of view of osteoblast differentiation and activity, this model of total absence of lamin A/C showed major similarities with a model of prelamin A accumulation [10] in terms of changes in osteoblast number and *ex vivo* differentiation of bone marrow precursors, therefore suggesting that the defects in osteoblast differentiation in both models are mostly associated with low levels of lamin A/C independently of the accumulation of prelamin A observed in the *Zmpste*24^−/−^ mice. Further studies testing this hypothesis are still required.

Considering that Runx2 is the most important regulator of OCN and OPN transcription [31] and that lamin A/C knockdown affects the nuclear binding capacity of the Runx2 complex *in vitro* [7], we then attempted to identify whether osteoblast differentiation is affected by the same mechanism *in vivo*. To test this hypothesis, we first looked at levels of Runx2 expression and transcription in *Lmna* ^−/−^ mice and WT controls. We found that expression and transcription of Runx2 in non-hematopoietic adherent stromal cells is not affected by the absence of lamin A/C, suggesting that, as *in vitro*, a similar mechanism involving the deficient nuclear binding of the Runx2 complex could constitute the underlying mechanism.

Lamin A/C is in close interaction with multiple nuclear proteins of the nuclear envelope that act as regulators of cell growth and differentiation. Although the bone defects observed in severe laminopathies such as HGPS could be also associated with accumulation of prelamin A in the presence of normal lamin C expression [19], for the purposes of this study we tested the role of the inner nuclear protein MAN-1 as one of the potential explanations for the limited capacity of the Runx2 complex to bind the nucleus and initiate osteogenic transcription in the absence of lamin A/C. The reasons for the selection of MAN-1 as our target protein have been explained above. The most important reason is the reported association between mutations in MAN-1 and hyperostosis syndromes, which may be explained by higher availability of Smads and Runx2 within the nucleus in the absence of MAN-1 thus facilitating their interaction and increasing bone formation [16]. In this case, we hypothesized that in the absence of lamin A/C, which physically interacts and regulates MAN-1 activity in the nucleus [15], Runx2 changes its localization and availability therefore affecting its capacity to interact and to form the Runx2 nuclear binding complex, decreasing OCN and OPN transcription and thus affecting osteoblastogenesis.

To test this hypothesis, we initially assessed the changes in MAN-1 expression in nuclear extracts obtained from *Lmna* ^−/−^ mice and WT controls. Bone marrow cells from *Lmna* ^−/−^ mice showed higher levels of MAN-1 protein expression. These high levels of MAN-1 expression could be explained by either potentially enhanced transcription or increased protein stability of MAN-1 induced by absence of lamin A/C. Overall, additionally to the already known physical interaction between lamin A/C and MAN-1 [15], our data suggest a new role of lamin A/C in the regulation of MAN-1 protein expression that should be explored in future studies.

Subsequently, we tested nuclear binding capacity of the Runx2 complex. As our *in vitro* experiments [7], Runx2 DNA binding activity was significantly reduced in absence of lamin A/C. This reduced binding activity could also be associated with either high levels of MAN-1 expression or by higher levels of free MAN-1, which could happen after losing its physical interaction with lamin A/C [15]. As in other models [32], [33], high levels of MAN-1 will inhibit the formation and translocation of osteogenic transcription complexes that interact with Runx2 in the nucleus. We then focused on the potential interaction between high levels of MAN-1 and Runx2 in the nucleus. Using colocalization we documented that, in absence of lamin A/C, Runx2 and MAN-1 share a similar localization in the nucleus of marrow precursors obtained from *Lmna* ^−/−^ mice.

In summary, we have demonstrated that lamin A/C is required for normal bone turnover and bone metabolism. Mice lacking lamin A/C show the typical features of senile osteoporosis. Although this bone phenotype could be a direct consequence of the mutation, other alterations (cardiovascular, neuromuscular, fat and hematopoiesis) may also be involved and deserve further exploration. Nevertheless and taken together with previous *in vitro* and *in vivo* studies, this evidence allows us to postulate that a decrease in lamin A/C could be a determinant player in the pathogenesis of age-related bone loss.

In conclusion, considering that levels of lamin A expression in bone progressively decrease with aging [23], it is tempting to propose that age-related changes in both the levels of lamin A/C expression by MSC and in the interaction between proteins of the nuclear lamina are associated with the key features of senile osteoporosis and could offer a new potential target to prevent bone loss thus maintaining appropriate levels of bone formation and bone mass.

## Materials and Methods

### Ethics statement

All steps were taken to ameliorate suffering in all work involving our study animals. The institutional Animal Ethics Committee at the Victor Chang Cardiac Research Institute (Ref. No. #07/11) approved all protocols, including those referring to analgesia and manipulation of animals.

### Animals

*Lmna* knockout mice in a C57Bl6x129Sv genetic background were generated as described [11]. Mice were genotyped by PCR amplification of tail DNA. Mutant male mice (^−/−^) and WT male littermates (n = 10 per group). To measure bone formation rates, mice were injected ip with 30 µg/g body weight of tetracycline 7 and 2 d before they were killed. Mice were sacrificed at 4 weeks of age. Both side tibiae and femora were obtained for further analysis. All experiments and measurements were repeated at least three times.

### Quantitative Radiologic Imaging

Micro-CT was performed in total body and femur of *Lmna* ^−/−^ mice and WT controls as previously described [10]. For femur analysis, after removal of soft tissues and overnight fixation in 4% paraformaldehyde right femur were analyzed using a Skyscan 1172 instrument (Skyscan, Antwerp, Belgium) equipped with a 1.3Mp camera was used to capture 2D serial cross-sections, which were used to reconstruct 3-dimensional images for the quantification of the volume of bone in both the diaphysis and distal metaphysis. Analyses of the bone microarchitecture were carried out in a region of interest (ROI), which was defined as the cancellous bone compartment beginning 0.6 mm proximal to the most proximal point of the growth plate and extending proximally 1.0 mm, corresponding to approximately 1.55 mm thick region of the distal femora. Nomenclature and abbreviations of micro-CT parameters follow the recommendations of the American Society of Bone and Mineral Research [34].

### Histology and Histomorphometry

For histomorphometric and histology analyses the left femur and tibia were fixed overnight in 4% paraformaldehyde, rinsed in 3 changes of PBS and embedded in polymethylmethacrylate (MMA) or a mixture of 50% MMA and 50% glycolmethacrylate (GMA). Serial 4- to 6-µm sections of MMA-embedded tissues were left unstained or stained with von Kossa, while 4-µm MMA-GMA decalcified sections were stained for alkaline phosphatase (ALP) (osteoblasts), toluidine blue (osteocytes), and tartrate resistance acid phosphatase (TRAP) (osteoclasts) activity as described previously [24]. Images were captured at two different magnifications (40× and 100×) using a Nikon Eclipse E100 microscope (Nikon Instruments Inc., Melville, NY, USA) and the primary histomorphometric data obtained using Bioquant Nova Prime image analysis software (Bioquant Image Analysis Corp, Nashville, Tennessee). Nomenclature and abbreviations conform to those recommended by the American Society for Bone and Mineral Research [35]. Histodynamic parameters MS/BS, MAR and BFR/BS were measured on the same specimens as above, using six representative fields per bone sample, in epifluorescent light.

### Serum Biochemistry and Bone Biomarkers

Serum concentrations of bone biomarkers and calciotropic hormones were analyzed at the Ageing Bone Research Centre-Nepean using commercial assays for serum C-Tx (ImmunoDiagnostic Systems Ltd, UK), P1NP (ImmunoDiagnostic Systems Ltd, UK), PTH (Immunotopics Inc San Clemente, CA, USA) and 25(OH)D (ImmunoDiagnostic Systems Ltd, UK).

### *Ex-vivo* cultures of bone marrow cells

To establish adherent bone marrow cultures, bone marrow cells were obtained and induced to differentiate into osteoblast as previously described [36]. Briefly, tibiae from four week-old *Lmna* ^−/−^ and ^+/−^ and their WT controls (*n* = 10 per group) were flushed using a 21-gauge needle attached to a 10 ml syringe filled with Dulbecco's modified Eagle's Medium (DMEM) (GIBCO BRL, Gaithersburg, MD, USA). Cells were filtered through a cell strainer with 70-micron nylon mesh (BD Bioscience, Bedford, MA, USA) and then combined to produce a volume of 2 ml containing ∼10^7^ cells/ml. Six-well plate cultures were then established in triplicate, with each well containing a 100-µl aliquot of cell suspension combined with 4 ml of fresh -MEM medium. The cells were incubated in MSC growth media at 37°C with 5% humidified CO_2_ and isolated by their adherence to tissue culture plastic. Medium was aspirated and replaced with fresh medium to remove non-adherent cells every 2 to 3 days. The adherent MSC were grown to ∼80% confluence for about 7 days defined as MSC at passage 0, harvested with 0.25% trypsin and 1 mM EDTA for 5 min at 37°C, diluted 1∶3 in MSC growth media, plated and grown to confluence for further expansion. After 2^nd^ and 3^rd^ passages, MSC were used for subsequent experiments.

To induce differentiation, a total of 10^4^ cells were diluted in osteogenic medium (prepared with DMEM, 10% FCS, 0.2 mM dexamethasone, 10 mmol/L β glycerol phosphate and 50 µg/mL ascorbic acid) and plated in six well plates. Media was aspirated and replaced with fresh osteogenic medium every 3 days. After 21 days in culture, cells were washed with PBS, ethanol fixed, stained for alkaline phosphatase, and counterstained with hematoxylin (Sigma). The colonies with more than 10% of cells staining positive for ALP were considered as colony forming units-osteoblasts (CFU-OB). Two independent observers quantified the number of CFU-OB using Nikon Eclipse E100 microscope (Nikon Instruments Inc., Melville, NY, USA).

### Semi-quantitative real time-polymerase chain reaction (RT-PCR)

Bone marrow cells were flushed and isolated as previously described. Total RNA was extracted from marrow cells using a QIAGEN RNeasy Mini extraction kit following manufacturer's instructions (QIAGEN Pty, Doncaster, VIC, Australia; cat# 74104). First strand complementary DNA (cDNA) synthesis was performed using 200 ng of total RNA, 50 ng random hexamers and 50 units reverse transcriptase at 42°C for 1 hour, as described by manufacturer (Bioline Australia Pty, Alexandria, NSW, Australia; cat# BIO-65025). Real-time PCR for expressed genes as markers for osteogenesis and Lamin A/C was performed in duplicate in a total reaction volume of 25 µl, 10% of which was cDNA (or water for non-template control), 3 mM MgCl_2_ and 250 nM of each forward and reverse specific primer for target genes and normalizer. Primers for specific detection of mRNA expression included: *Lmna* (F: 5′-tgagtacaacctgcgctcac-3′; R: 5′-tgactaggttgtccccgaag-3′); runt-related transcriptional factor 2 (RUNX2) (F: 5′-gccgggaatgatgagaacta-3′, R: 5′-ggaccgtccactgtcacttt-3′); OPN (F: 5′-tgcacccagatcctatagcc-3′, R: 5′-ctccatcgtcatcatcatcg-3′); OCN (F: 5′-cttggtgcacacctagcaga-3′; 5′-accttattgccctcctgctt-3′). All PCRs were performed in a Corbett Rotor-Gene™ 3000 (QIAGEN Pty) using SYBR green with no-ROX reaction mix and a standard thermal profile as described by supplier (Bioline Australia Pty, Alexandria, NSW, Australia; cat# QT6750-02). Quantitative RT-PCR data was defined by threshold cycle (Ct) normalized for the housekeeping gene glyceraldehyde-3-phosphate dehydrogenase (GAPDH) (F: 5′-aactttggcattgtggaagg-3′, R: 5′-acacattgggggtaggaaca-3′).

### Western blot analysis

For western blot analysis, 20 µg of total protein were loaded on polyacrylamide gels and separated by standard SDS-polyacrylamide gel electrophoresis. To control for differences in gel migration, exposure time, antibody incubation etc, samples were run on the same gels and transferred to the same PVDF membranes (Amersham, UK). Blots were blocked overnight in 2.5% non-fat dried milk and probed with antibodies directed against Lamin A/C, Runx2, OPN, MAN-1, and OCN (1∶1000, Santa Cruz Biotechnology, Santa Cruz, CA, USA). Positive controls were included in all experiments as provided by the manufacturer (Santa Cruz Biotechnology, Santa Cruz, CA, USA, USA), to confirm antibody specificity. Secondary antibodies conjugated to horseradish peroxidase were from Sigma (1∶5000). Antigen-antibody complexes were detected by chemiluminescence using a kit of reagents from ECL (Amersham, UK) and blots were exposed to high-performance chemiluminescence film (Amersham, UK). Films were scanned and the optical density of each specific band analysed using the ImageMaster program and expressed as OD/mm2/100 µg of total protein. The signals were quantified by densitometry and normalized according to Tubulin density.

### Runx2 activity measurement

Active Runx2 binding to DNA was determined using the ELISA-based Runx2 activation TransAM™ kit (Active Motif, Rixensart, Belgium) as previously described [7], [24]. The Trans-AM Runx2 Kit contains a 96-well plate on which an oligonucleotide containing a Runx2 consensus-binding site (5′-AACCACA-3′) has been immobilized. The active form of Runx2 contained in nuclear extract specifically binds to this oligonucleotide. The primary antibody used in the Trans-AM Runx2 Kit recognizes an accessible epitope on Runx2 protein upon DNA binding. Addition of a secondary horseradish peroxidase (HRP)-conjugated antibody provides a sensitive colorimetric readout easily quantified by spectophotometry (450 nm). To quantify active Runx2 binding, 15–20 mg of nuclear extract was measured using the Trans-AM Runx2 Kit according to the manufacturer's instructions (Active Motif, Carlsbad, CA, USA).

### Confocal microscopy

To determine the intranuclear distribution of Runx2 and MAN-1, bone marrow cells were flushed and plated in two-well slides containing MSC growth media as previously described. After passage 2, cells were fixed with 95% methanol for 15 min and incubated with MAN-1 or Runx2 antibodies (1∶500) followed by a rhodamine- or fluorescein-conjugated secondary antibody, respectively. DAPI was used for nuclear counterstaining. Sections were mounted using Vectashield Hard Set mounting medium with 4′,6-diamidino-2-phenylindole (Burlingame, CA) and viewed using an LSM 510 Zeiss confocal microscope (Carl Zeiss, Thornwood, NY) at 40× and 100× magnification.

### Statistical Analysis

Results are expressed as means ± SD and differences between groups of mice were determined by the Student *t* test and the Mann-Whitney test. A *P* value of ≤0.05 was considered statistically significant.
